# Healthy Eating Index and Bone in Obesity

**DOI:** 10.1007/s11914-026-00995-w

**Published:** 2026-11-03

**Authors:** Deeptha Sukumar, E. A. Alsayed

**Affiliations:** 1 Department of Health Sciences- Nutrition Division, College of Nursing and Health Professions, Drexel University, Philadelphia, 19102 USA; 2 Department of Clinical Nutrition, College of Nursing and Health Sciences, Jazan University, Jazan, 45142 Saudi Arabia

**Keywords:** Healthy Eating Index, Obesity, Bone Quality, Bone Density, Dietary Quality, Diet Quality

## Abstract

**Purpose of Review:**

Individuals living with overweight or obesity have a higher bone mineral density, however bone quality may be compromised, leading to an increased susceptibility to fractures and impaired skeletal integrity. Nutrition is a potentially modifiable factor that may influence both body weight and bone metabolism through effects on inflammation, body composition, and metabolic regulation. In particular, dietary quality indices such as the Healthy Eating Index (HEI) provide a comprehensive assessment of adherence to dietary guidelines and may offer insight into how overall eating patterns influence bone quality among individuals with excess body weight.

**Recent Findings:**

In this review, we have summarized the impact of dietary quality using the HEI on bone health in individuals who are living with overweight or obesity. Recent studies show a positive relationship of high HEI with bone parameters in overweight and obese individuals. Specifically, studies report on a higher Trabecular bone score (TBS), lumbar and femoral neck BMD, lower osteoporosis risk and higher serum 25 hydroxy vitamin D levels in populations with a higher HEI.

**Summary:**

Currently studies assessing impact of HEI on bone health in obesity are cross sectional in nature. Future studies should assess the impact of increasing HEI on bone health in a prospective design and the impact of weight loss and increasing HEI on bone quality in individuals living with overweight and obesity.

## Introduction

The relationship between obesity and bone health is increasingly recognized as complex, dynamic, and paradoxical. Traditionally, higher body weight was believed to exert a protective effect on the skeletal system because greater mechanical loading was associated with increased bone mineral density (BMD). However, advances in imaging technologies and methods for assessing bone microarchitecture and strength have challenged this long-standing assumption. Emerging evidence suggests that although individuals with overweight or obesity often present with higher BMD, bone quality may nevertheless be compromised, leading to an increased susceptibility to fractures and impaired skeletal integrity [1, 2].

Bone quality encompasses factors such as bone microarchitecture, turnover, mineralization, and structural strength, all of which contribute to overall skeletal resilience. In individuals with obesity, excess adiposity may adversely affect these characteristics through chronic low-grade inflammation, altered adipokine secretion, insulin resistance, and disruptions in endocrine pathways that regulate bone remodeling [1, 2]. Despite growing recognition of these biological mechanisms, the role of dietary patterns and overall diet quality in mediating the obesity–bone health relationship remains less clearly understood. Nutrition is a potentially modifiable factor that may influence both obesity and bone metabolism through effects on inflammation, nutrient availability, body composition, and metabolic regulation. In particular, dietary quality indices such as the Healthy Eating Index (HEI) provide a comprehensive assessment of adherence to dietary guidelines [3] and may offer insight into how overall eating patterns influence bone quality among individuals with excess body weight.

In this review, we will examine the role of dietary quality measured using the Healthy Eating Index (HEI) on bone health in individuals who are overweight or living with obesity. By exploring the role of diet in the interplay among obesity, endocrine regulation, and bone quality, this review aims to provide a more comprehensive understanding of dietary factors that may contribute to compromised bone integrity in populations with excess adiposity.

## What is the Healthy Eating Index?

The Healthy Eating Index (HEI) is a measure of diet quality used to assess how well a set of foods aligns with key recommendations and dietary patterns published in the Dietary Guidelines for Americans. It was originally developed in the year 1995 and revised in 2005, 2015 and the current version of HEI is the HEI-2020. The HEI-2020 aligns with the 2020-25 Dietary Guidelines for Americans and consists of a total of 13 components, including nine adequacy components and four moderation components as reflected in the Dietary Guidelines for Americans [3]. The adequacy components promote the consumption of beneficial food groups, such as seafood, plant protein foods, whole grains, dairy, vegetables and fruits. In contrast, the moderation components aim to restrict the consumption of less healthy dietary elements, including sodium, saturated fats, added sugars, and refined grains. The HEI uses a scoring system from 0 to 100 to evaluate foods and food groups across 13 different components, and a score of 100 reflects perfect alignment with the dietary guidelines [4]. A low score reflects a poor dietary quality, while a higher score (typically in the ranges of 80 and above) reflects a satisfactory dietary quality (USDA). A higher HEI score is consistently associated with improved overall health outcomes [5]. Individuals with higher HEI tend to have a lower risk of chronic diseases such as cardiovascular disease, diabetes, and obesity [6–8]. Adherence to dietary guidelines reflected by a high HEI score also supports better metabolic and immune function [9]. Previous studies have shown that healthier eating patterns contribute to improved physical and mental well-being [10]. Moreover, higher HEI scores are linked with reduced mortality risk and enhanced quality of life across diverse populations [5].

## Dietary Quality in Obesity

Previous studies have reported that a high dietary quality is associated with a lower BMI and better metabolic outcomes in the individuals living with overweight or obesity. In the multiethnic cohort study that examined the association of diet quality with BMI at 3 time points among 1860 participants, it was found that HEI-2015 was associated with lower probability of overweight and obesity across different ethnic groups, especially in individuals’ midlife to older age [11]. The Nambour Skin Cancer Study, a 15-year longitudinal study in an Australian population comprising 1231 adults aged 25–75 years, found that men but not women with the highest diet quality, measured using the Australian Dietary Guideline Index (DGI), had the lowest gain in BMI per year [12]. Furthermore, the Resilience for Eating and Activity Despite Inequality study that assessed dietary quality using the DGI in women found that a 1-point change in the DGI was inversely associated with BMI gain across 3 time points [13]. In the prospective EPIC-PANACEA project, an improvement of Mediterranean diet score reduced the likelihood of developing overweight or obesity by 10% over 5 years [14]. Collectively, these findings provide strong evidence that dietary quality is a key predictor of long-term BMI and weight gain across the lifespan.

## Dietary Factors and Bone Health- Mechanisms

The HEI encourages the consumption of adequacy components i.e., consumption of beneficial food groups, such as seafoods, plant protein foods, whole grains, dairy, vegetables, and fruits. In contrast, the moderation components aim to restrict the consumption of less healthy dietary elements, including sodium, saturated fats, added sugars, and refined grains. In this section, we will discuss the impact of HEI adequacy components and moderation components on bone health.

### Adequacy Components

Protein is a critical component of bone and makes up about half of the bone volume and about a third of its mass. Adequate dietary protein is critical for optimal bone mass throughout the lifespan. Several Randomized Controlled Trials (RCT) and cohort studies underscore the importance of protein on bone health. Collectively, these studies suggest positive trends on BMD at most bone sites [4]. Whole grains provide vital bone-building minerals and impact inflammatory pathways, although high intakes of whole grains may be associated with reduced absorption of minerals. Epidemiological studies also show a positive association between intake of whole grains and bone health [15] and an inverse association of whole grain intake with hip fracture [16, 17]. Dairy products have shown to positively impact bone accrual during growth and bone turnover in adults. Fermented dairy, with its added probiotic potential and improved minerals, appears to be associated with a lower risk of hip fracture in older adults [18]. Phytochemicals, vitamins, and minerals in fruits and vegetables may have a positive impact on BMD. Findings from 10 cohort studies in over 500,000 individuals revealed with moderate certainty that a greater consumption of vegetables only might reduce the risk of fracture; however, this was not true with total fruit and vegetable or only fruit intake [19].

### Moderation Components

High dietary sodium intake has shown to increase urinary calcium excretion, and reducing sodium intake can lower urinary calcium excretion. A recent meta-analysis of 6 articles showed that high sodium diets significantly increased urinary excretion of calcium in women after menopause, with a pooled mean difference of 29.38 mg/day [20]. Findings from the Women’s Health Initiative, a large, prospective, observational cohort study of 69,735 postmenopausal women, showed sodium intake at or near recommended levels was not likely to impact bone metabolism [21]. However, excess salt intake (Mean intakes of 8.5 g/day) has shown to reduce bone density independently of age and lifestyle-related diseases in the general female population [22]. Saturated fats have also shown to negatively impact bone health in experimental animal models [23, 24]. Findings from the NHANES III cohort showed that saturated fat intake was negatively associated with femoral neck BMD among men < 50 y old [25]. The Young Finns study also noted higher odds of fractures in the higher compared with the lowest tertiles of saturated fatty acid (SFA) intakes, independently of many risk factors of osteoporosis [26]. The impact of added sugars, especially sugar-sweetened beverages (SSB), on bone health has been addressed in multiple previous studies. Studies show a positive association of high intakes of SSB with increased bone fragility, increased all-cause mortality among individuals with compromised bone health, and an inverse relationship with BMD [27, 28]. The consumption of refined grains has also shown to be positively associated with a risk of osteoporosis [29].

## Relationship of Healthy Eating Index with Bone Quality in Obesity

**Fig. 1:**
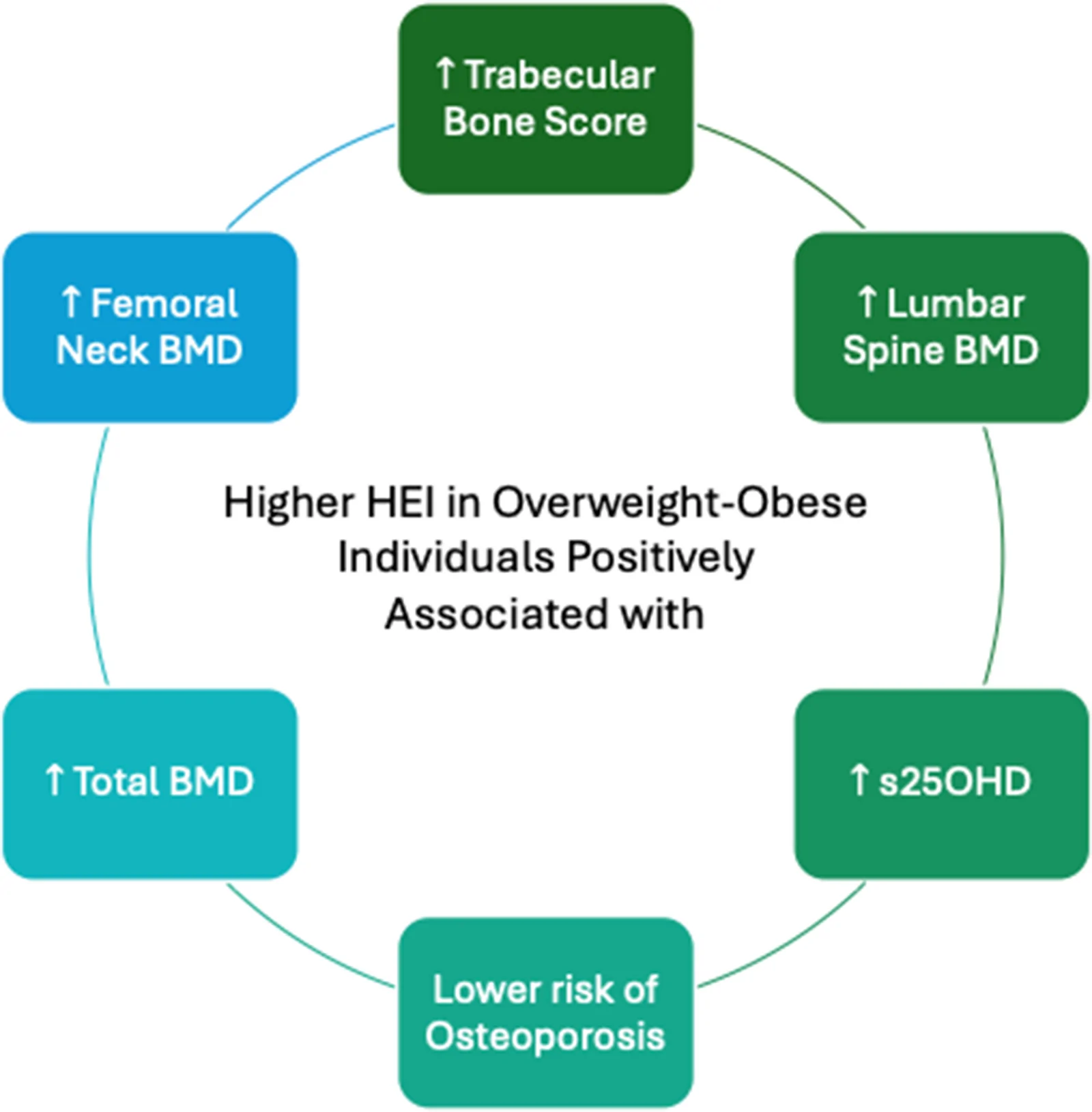
Relationship of HEI with bone health parameters in individuals living with overweight and obesity [30–35]. HEI: Health Eating Index, BMD: Bone Mineral Density, aBMD: Areal Bone Mineral Density, TBS: Trabecular Bone Score, s25OHD: Serum 25 hydroxy Vitamin D

The relationship between HEI and bone quality has been assessed in several populations with a mean BMI > 25 kg/m^2^. This narrative review was conducted to integrate current evidence on the relationship between dietary quality, specifically assessed using HEI and bone health in overweight and obese individuals. A comprehensive literature search was conducted using PubMed/MEDLINE, Cochrane Library, CINAHL, Scopus, and Web of Science, covering studies published up to June 15, 2026. The following keywords were used: “Healthy Eating Index” AND “Bone” AND “Obesity” OR “HEI” AND “Obesity” AND “Bone”. Additionally, reference sections of included articles were manually screened for relevant studies. Eligible studies met the following criteria: (1) original research articles, systematic reviews, meta-analyses, or letters to the editor; (2) reported on HEI, bone health outcomes; and (3) were published in English with full-text availability. Only studies that reported outcomes related to bone health such as BMD, osteoporosis incidence, fracture risk, and BMI or body weight were included in the summary below. A summary of these studies is presented in Table 1 and the impact of HEI on bone health is illustrated in Fig. 1.

**Table 1:** Studies that examine association of HEI with bone health in overweight and obese populations

Authors, Year	Participant, Demographics (*N*, Age-y), BMI- kg/m2)	HEI version/ Mean HEI	Bone variables	Main findings
Yu H and Liu Y 2026	*N* = 7007 Age 47.2 ± 0.5 BMI 28.17 ± 0.13	HEI-2020 50.86 ± 0.35	Trabecular Bone Score (TBS)	Positive association between HEI and TBS, with strongest association in males with obesity.
Wang K 2024	*N* = 3421 Age: 62.63 ± 8.85 38.9% were obese	HEI-2015 57.72 ± 13.0	Osteoporosis Risk, BMD	Higher tertiles of HEI associated with low osteoporosis risk, no association with BMD
Babazadeh-Anvigh B 2020	*N* = 416 Age: 48.0 ± 12.4 BMI: 27.1 ± 4.4	HEI-2015 70.4 ± 12.4	BMD Osteoporosis Risk	Premenopausal women: Higher HEI was associated with higher FN-BMD, lower risk of LS osteoporosis Postmenopausal women: Higher HEI was associated with higher lumbar spine BMD, femoral neck BMD and had a lower risk of osteoporosis at femoral neck
Ghadiri M 2023	*N* = 131 Age: 57.2 BMI: 29.5	HEI-2015 NA	Femoral Neck BMD, Lumbar spine BMD	Participants in the highest tertile of HEI had higher femoral and lumbar BMD.
Kindler JM 2021	*N* = 415 (*N* = 128 with obesity) Age: 18.5 ± 3.5 BMI Z Score: 2.33	HEI-2005 46.6	Whole body aBMD	Significant positive association between HEI and aBMD Z-score. Whole grains and empty calories were positively associated with aBMD Z-score.
Suhett L 2023	*N* = 122 Age: 37.9 ± 12.7 BMI: 30.0 ± 4.6	HEI-2015 54.42 ± 16.25	BMD, Bone regulating hormones	Positive association between HEI scores and s25OHD in adults who were overweight or obese with Metabolically unhealthy obese phenotype

Abbreviations: *HEI* Health Eating Index, *BMI* Body Mass Index, *BMD* Bone Mineral Density, *aBMD* Areal Bone Mineral Density, *TBS* Trabecular Bone Score, *s25OHD* Serum 25 hydroxy Vitamin D

### HEI and Trabecular Bone Score in Adults Living with Overweight and Obesity

Trabecular bone score (TBS) reflects trabecular microarchitecture and predicts fracture risk. The study by Yu and Liu et al. [30] analyzed the relationship between HEI and TBS using HEI-2020 in 7,007 adults (3,449 males and 3,558 females), with a mean age of 47.19 years and mean BMI of 28.17 kg/m2. The mean HEI-2020 score in the overall population was 50.86 ± 0.35. Higher HEI-2020 scores were positively associated with TBS and participants in the highest HEI-2020 tertile (Mean HEI of 64.54) had a significantly higher TBS compared with those in the lowest tertile (Mean HEI of 38.79). Those in the high-HEI cluster also had higher TBS values compared to the low-HEI cluster (Yu & Liu, 2026). Interestingly, BMI modified the HEI-2020 TBS association; the association was non-significant in normal-weight and overweight individuals but was significant only among those with obesity. Also, the association between HEI-2020 and TBS was strongest in males with obesity, weaker but marginally significant in females with obesity, and not significant in males without obesity or females without obesity.

### HEI and Bone Density in Women living with Overweight and Obesity

Using data from NHANES 2007–2018, Wang et al. [31] analyzed the associations between HEI, bone mineral density, and the prevalence of osteoporosis in 3421 postmenopausal women. The mean age of these women was 63 years, and 38.9% of these women were in the obese category. Women in the higher tertiles of HEI had a lower risk of osteoporosis, while no significant relationship between HEI-2015 and the risk of low BMD was demonstrated. The results suggest that as HEI-2015 increased, osteoporosis risk decreased in a nonlinear manner; specifically, total vegetables, refined grains, and greens and beans were identified as components that contributed the most to reducing the osteoporosis risk. Higher intake of total vegetables, greens and beans, total fruits, whole fruits, and whole grains was associated with decreased odds of osteoporosis among elderly adults. Surprisingly saturated fat intake was also protective against osteoporosis in this population. Although over 1/3rd of women were in the obese category, no specific observations were made specifically on HEI’s contribution to BMD and osteoporosis risk in this subgroup.

Another study in women in Iran analyzed the associations of HEI with BMD according to menopausal status [32]. These women had a mean age of 48, BMI 27 Kg/m^2^ and HEI was calculated using HEI-2015. Study findings showed that premenopausal women had a lower HEI-2015 score (67.4 vs. 75.8) than postmenopausal women. Among premenopausal women, those in the highest tertile of the HEI-2015 score had higher femoral neck BMD, BMI and a lower risk of lumbar spine osteoporosis than those in the lowest tertile of HEI. Among postmenopausal women, those in the highest tertile of the HEI-2015 score had higher lumbar spine BMD and femoral neck BMD and had a lower risk of osteoporosis at the femoral neck. Interestingly, premenopausal women with a higher HEI-2015 score had a higher BMI, while this was not observed in the postmenopausal women. Mean BMI in both pre- and postmenopausal women was in the overweight range; however, no specific observations were made specifically on HEI’s contribution to BMD and osteoporosis risk in this subgroup of overweight women.

A case-control study [33] examined 131 postmenopausal women with osteoporosis and 131 healthy postmenopausal women with a mean age of 57 years and BMI of 29 kg/m^2^. Findings showed that participants in the highest tertile of HEI with higher intakes of whole grains, fruits, vegetables, nuts, legumes, and dairy were likely to have higher femoral and lumbar BMD.

### Other Studies that Examine Relationship of HEI with Bone Health

In another study in young adults the association of HEI and bone health was similar, with HEI being positively associated with aBMD [34]. The study in 415 young adults with a mean age of 18 years included 3 groups of participants- normal weight, obese and those with type 2 diabetes. Although HEI was associated with aBMD in the entire cohort, there were overall null associations between HEI and aBMD in analyses including only the obesity and type 2 diabetes groups. Total HEI and subcomponent scores were not significantly associated with aBMD Z-score in the obesity and type 2 diabetes group.

The association between HEI-2015, bone density, and bone-regulating hormones was assessed in a study [35] in 122 adults who were overweight or obese and further classified as metabolically healthy obese (MHO) or metabolically unhealthy obese (MUO), based on the prevalence of metabolic syndrome risk factors. HEI scores were positively associated with serum 25hydroxy vitamin D (s25OHD) levels in the MUO phenotype group only, and dietary score components, such as fruits, seafood and plant protein, added sugars, whole grains, and fatty acids, were associated with specific serum bone health markers. However, HEI scores were not associated with BMD measures, nor with other bone-regulating hormones and turnover markers in this study.

## Conclusions

Alterations in the endocrine milieu, body composition, and increased production of pro-inflammatory cytokines contribute to compromised bone quality in obesity. This review highlights how dietary quality also plays a crucial role in maintaining bone health and optimizing bone quality among individuals with obesity. Based on recent studies, higher HEI scores, which reflect greater adherence to dietary guidelines and overall diet quality, have been consistently associated with superior bone quality and skeletal outcomes in overweight and obese populations. Although obesity-related factors, including endocrine dysregulation, altered body composition, and a chronic pro-inflammatory state, may adversely influence bone metabolism and contribute to skeletal fragility, dietary factors represent important and potentially modifiable determinants of bone health. Adequate intake of bone-supportive nutrients, coupled with adherence to healthy dietary patterns reflected by a high HEI, may reduce inflammation and oxidative stress and support the maintenance of bone microarchitecture.

The current evidence of the impact of HEI on bone health however is largely restricted to observational studies. Few, if any prospective studies are designed specifically to assess the impact of changing HEI overtime on bone health. Randomized controlled trials that are specifically designed to improve overall HEI, should assess the impact of such intervention on BMD and bone quality in individuals living with overweight and obesity. Furthermore, the impact of dietary weight loss interventions with a focus on increasing HEI and its impact on bone quality should also be assessed in individuals living with overweight and obesity and specifically in postmenopausal women. Consequently, dietary interventions targeted at improving overall dietary quality, rather than single macro or micronutrient modifications, should be integrated into obesity management approaches to optimize bone quality and reduce the risk of osteoporosis and fractures across the lifespan.

## Key References

- Suhett LG, Cheung M, Dall RD, Sukumar D. Healthy eating index and bone health markers in adults with metabolically healthy and unhealthy obese phenotypes. Hum Nutr Metab. 2023 Jun;32:200186. doi: 10.1016/j.hnm.2023.200186

- ○ This review was inspired by findings from our laboratory in this Original Research study. We analyzed the relationship of HEI with bone density and markers of bone health in 122 adults living with obesity who were further categorized as Metabolically Healthy Obese or Metabolically Unhealthy Obese. Results showed a positive association between HEI scores and s25OHD in overweight or obese adults with Metabolically unhealthy obese phenotype.

- Babazadeh-Anvigh B, Abedi V, Heydari S, Karamati D, Babajafari S, Najafi A, Rashidkhani B, Shariati-Bafghi SE, Karamati M. Healthy eating index-2015 and bone mineral density among adult Iranian women. Arch Osteoporos. 2020 Oct 1;15(1):151. doi: 10.1007/s11657-020-00826-0.

- ○ Amongst 416 Iranian women, the association of HEI on bone parameters were analysed according to menopausal status. Among premenopausal women, those in the highest tertile of the HEI-2015 score had higher femoral neck BMD, BMI and a lower risk of lumbar spine osteoporosis than those in the lowest tertile of HEI. Among postmenopausal women, those in the highest tertile of the HEI-2015 score had higher lumbar spine BMD and femoral neck BMD and had a lower risk of osteoporosis at the femoral neck.

- Ghadiri M, Soltani M, Rajabzadeh-Dehkordi M, Gerami S, Shateri Z, Nouri M, Gargari BP. The relation between dietary quality and healthy eating index with bone mineral density in osteoporosis: a case-control study. BMC Musculoskelet Disord. 2023 Jul 18;24(1):584. doi: 10.1186/s12891-023-06704-3.

- ○ This is a case-control study that examined 131 postmenopausal women with osteoporosis and 131 healthy postmenopausal women. Findings showed that participants in the highest tertile of HEI with higher intakes of whole grains, fruits, vegetables, nuts, legumes, and dairy were likely to have higher femoral and lumbar BMD.

- Kindler JM, Gallo S, Khoury PR, Urbina EM, Zemel BS. Diet Quality and Bone Density in Youth with Healthy Weight, Obesity, and Type 2 Diabetes. Nutrients. 2021 Sep 21;13(9):3288. doi: 10.3390/nu13093288.

- ○ This is one of the only studies that examined the role of HEI on bone health in young adults and also included a population with obesity. Significant positive association between HEI and aBMD Z-score was observed amongst the youth population.

- Yu H, Liu Y. Healthy Eating Index-2020 and Trabecular Bone Score in US Adults: A Cross-Sectional Analysis of NHANES 2005-2008. J Am Nutr Assoc. 2026 Mar 4:1-12. doi: 10.1080/27697061.2026.2636544.

- ○ In one of the largest study that assessed the relationship of HEI with bone n over 7000 adults, HEI was assessed using HEI-2020 and higher HEI scores were positively associated with Trabecular Bone Score, an important marker of Bone quality. Interestingly the positive association between TBS and HEI was stronger in those with Obesity.

## Data Availability

No datasets were generated or analysed during the current study.

## Declarations

### AI Statement

The use of generative artificial intelligence was limited to assistive purposes such as rewriting certain parts of the text. All scientific content were conceived and verified by the authors.
